# Physiological meaning of bimodal tree growth-climate response patterns

**DOI:** 10.1007/s00484-024-02706-5

**Published:** 2024-05-30

**Authors:** Ulf Büntgen, Jan Esper

**Affiliations:** 1 Department of Geography, University of Cambridge, Cambridge, CB2 3EN UK; 2 Global Change Research Institute (CzechGlobe), Czech Academy of Sciences, Brno, 603 00 Czech Republic; 3 Department of Geography, Faculty of Science, Masaryk University, Brno, 611 37 Czech Republic; 4 Department of Geography, Johannes Gutenberg University, 55099 Mainz, Germany

**Keywords:** Climate change, Dendrochronology, Global warming, Forest ecology, Tree rings

## Abstract

Correlation coefficients are widely used to identify and quantify climate signals in proxy archives. Significant relationships between tree-ring chronologies and meteorological measurements are typically applied by dendroclimatologists to distinguish between more or less relevant climate variation for ring formation. While insignificant growth-climate correlations are usually found with cold season months, we argue that weak relationships with high summer temperatures not necessarily disprove their importance for xylogenesis. Here, we use maximum latewood density records from ten treeline sites between northern Scandinavia and southern Spain to demonstrate how monthly growth-climate correlations change from narrow unimodal to wide bimodal seasons when vegetation periods become longer and warmer. Statistically meaningful relationships occur when minimum temperatures exceed ‘biological zero’ at around 5° C. We conclude that the absence of evidence for statistical significance between tree growth and the warmest summer temperatures at Mediterranean sites is no evidence of absence for the physiological importance of high summer temperatures for ring formation. Since correlation should never be confused with causation, statistical values require mechanistic understanding, and different interpretations are needed for insignificant correlations within and outside the growing season.

**Supplementary Information:**

The online version contains supplementary material available at 10.1007/s00484-024-02706-5.

## Background and motivation

Since the first articles in tree-ring research were published more than 100 years ago (Kapteyn 1908, 1914; Douglass 1909, 1914), correlation coefficients are frequently used to identify and quantify growth-climate relationships in dendroclimatology (Schulman 1956; Fritts 1976; Cook and Kairiukstis 1990; Briffa et al. 2002). Most commonly used are Pearson’s correlation coefficients (Pearson 1895), which measure the strength and direction of a linear relationship between two variables. A correlation coefficient of 1 describes a perfectly positive relationship, whereas a value of -1 refers to a negative (inverse) relationship. Correlation coefficients at, or close to zero indicate no, or very weak relationships between two variables. The statistical significance of a correlation coefficient is usually defined by its *p*-value, which is calculated from the number of datapoints and the linear relationship between the variables. In dendroclimatology, *p*-values should be corrected for serial correlation structures (i.e., trends in timeseries that can be expressed by the first-order autocorrelation), as these reduce the degree of freedom (Trenberth 1984; Osborn and Briffa 2000). Although Pearson correlation coefficients cannot determine whether one of the variables is dependent on the other, nor what proportion of the variation in the dependent variable is attributable to the independent variable, correlation is often interpreted as causation.

In dendroclimatology (Fritts 1976), and most other fields of high-resolution paleoclimatology (Jones et al. 2009), significant positive correlations between proxy parameters and meteorological measurements are commonly understood as proxy-target dependency (e.g., St. George 2014), whereas insignificant correlations between predictor and predictand are usually interpreted in a way that climate variation is not important for proxy formation. Even more advanced timescale-dependent reconstruction techniques in tree ring-based high-resolution paleoclimatology rely on simple correlation coefficients (Guiot 1985; Osborn and Briffa 2000). The quality of dendro chronologies and their subsequent climate reconstructions is often defined and ranked by proxy-target correlations (Esper et al. 2016; Ljungqvist et al. 2020).

Here, we re-assess the climate response of ten maximum latewood density (MXD) records from near upper elevational treeline ecotones along a latitudinal gradient from northern Scandinavia to southern Spain. We use MXD rather than tree-ring width as the former normally reflects stronger temperature signals across wider ecological ranges and is less prone to biological memory (Esper et al. 2015). Our hypothesis is that insignificant correlation coefficients between tree-ring proxy and climate target data not only occur outside the growing season when trees are dormant, but also in the middle of long growing season when temperatures are so high that year-to-year variation in summer warmth is not impacting ring formation.

## Materials and methods

**Fig. 1:**
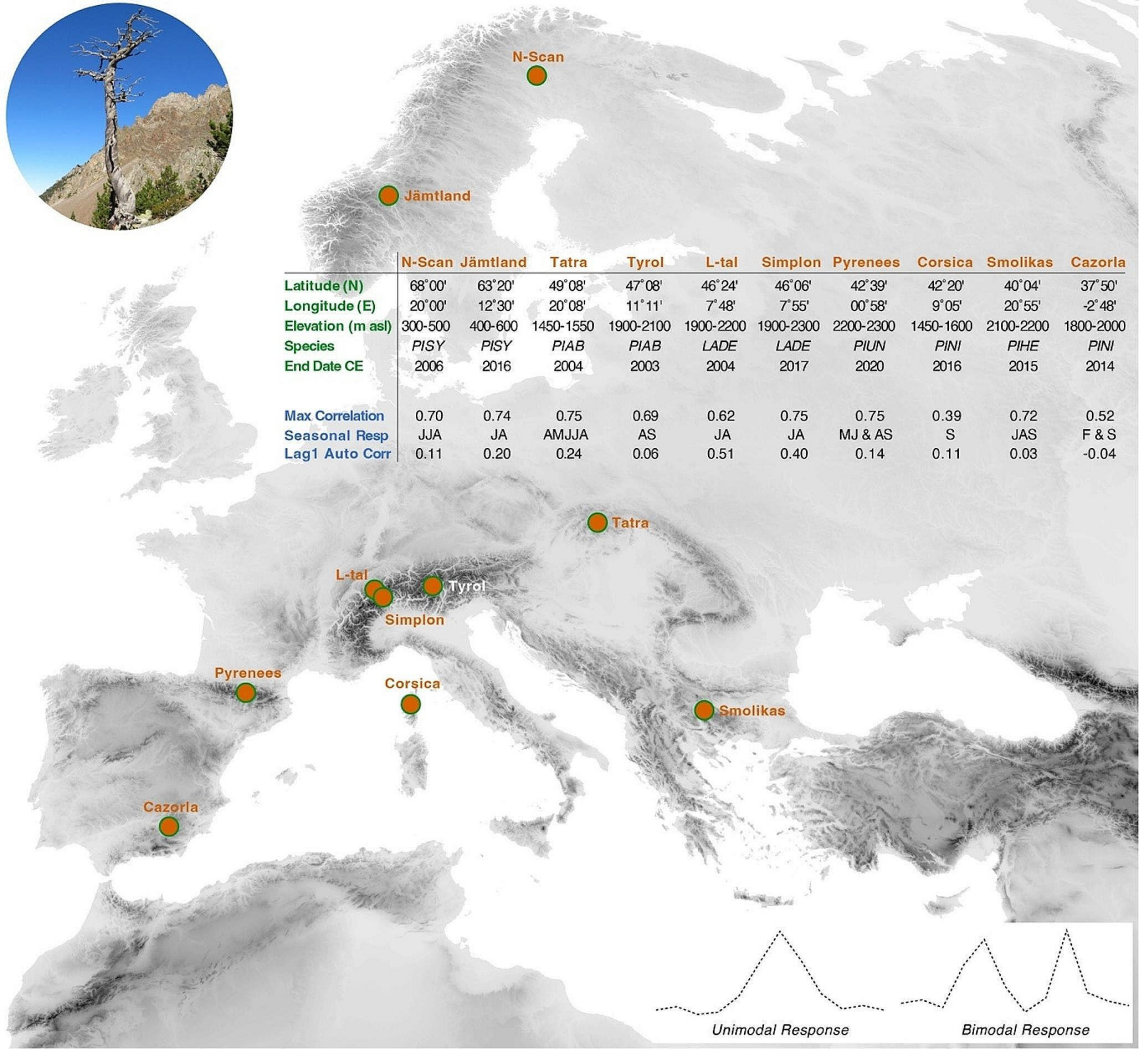
Location (background map) and description (inset table) of the ten maximum latewood density (MXD) records used in this study (see Figs. S1–S10 for details). JJA = June-August, JA = July-August, AMJJA = April-August, AS = August-September, MJ = May-June, S = September, JAS = July-September, F = February. The upper left picture shows a dry-dead mountain pine (*Pinus uncinata*) from the upper treeline in the central Spanish Pyrenees, and the lower right schematic graphs describe the concept of unimodal and bimodal growth-climate response patterns

We compiled ten MXD-based temperature reconstructions that have been published since 2006 for different regions in Europe (Fig. 1). Two Scots pine (*Pinus sylvestris*; PISY) datasets from northern and central Scandinavia represent treeline ecotones between 300 and 600 m asl and have recent end dates in 2006 and 2016 (Esper et al. 2012; Gunnarson et al. 2011 updated), respectively. Two spruce (*Picea abies*; PIAB) datasets from the Slovakian Tatra and the Austrian Alps represent treeline ecotones around 1500 and 2000 m asl and have recent end dates in 2004 and 2003 (Büntgen et al. 2007; Esper et al. 2007), respectively. Two larch (*Larix decidua*; LADE) datasets from the Swiss Alps represent treeline ecotones between 1900 and 2300 m asl and have recent end dates in 2004 and 2017 (Büntgen et al. 2006; Kuhl et al. 2024), respectively. One mountain pine (*Pinus uncinate*; PIUN) dataset from the Spanish Pyrenees represents treeline ecotones at 2200–2300 m asl and has its recent end date in 2020 (Büntgen et al. 2024). Two black pine (*Pinus nigra*; PINI) datasets from northern Corsica and the Cazorla mountains in southern Spain represent ecotones around 1550 and 1900 m asl and have recent end dates in 2016 and 2014 (Römer et al. 2021; Esper et al. 2020a), respectively. One Bosnian pine (*Pinus heldreichii*; PIHE) dataset from Mount Smolikas in northern Greece represents ecotones around 2150 m asl and has its recent end date in 2015 (Esper et al. 2020b).

We calculated Pearson correlation coefficients between the ten MXD records and monthly mean temperatures from January to December of ring formation. Minimum, mean and maximum temperatures were used to evaluate the importance of diurnal ranges and approximate thermal constrains on ring formation. For each tree-ring site (Fig. 1), we extracted observational temperature data from the nearest 0.25° x 0.25° grid box of the latest E-OBS product v28.0e that reaches back to 1920 CE (Cornes et al. 2018). Correlation coefficients were calculated over three different periods from 1920, 1950 and 1970 to 2003 that are common to all proxy-target combinations, as well as from 1920, 1950 and 1970 to the end dates of the individual MXD records ranging from 2003 to 2020 (Fig. 1). The standard deviation of all 18 *r* values per month was considered to express the strength of the obtained growth-climate response patterns.

## Results and discussion

**Fig. 2:**
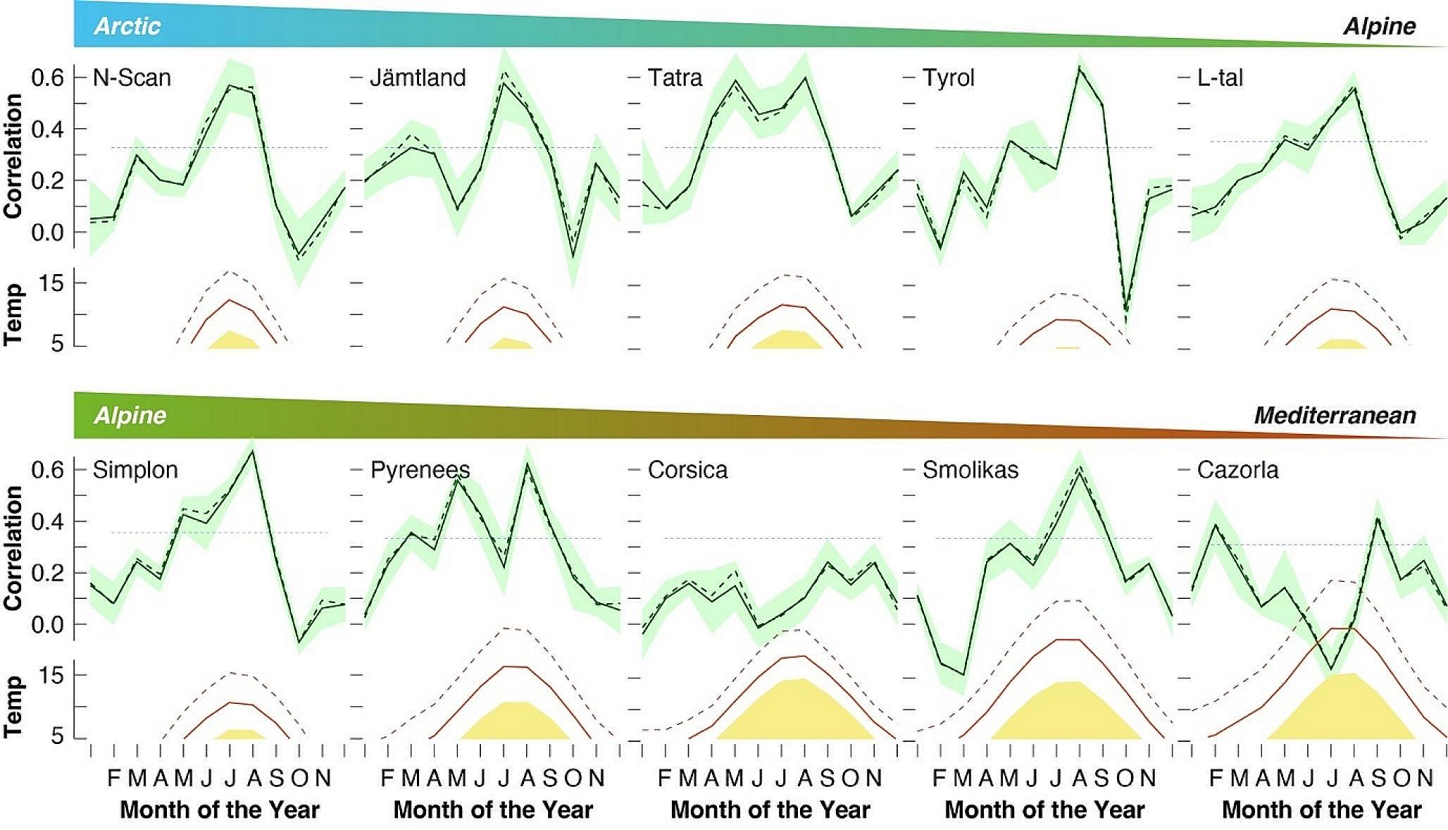
Pearson correlation coefficients between monthly temperature measurements and maximum latewood density (MXD) records from ten treeline sites between northern Scandinavia (upper left) and southern Spain (lower right) (Fig. 1). Solid/dashed lines refer to the mean/median of all correlation pairings between MXD and minimum, mean and maximum temperatures that were calculated over six different periods between 1920 and present (see Figs. S1–S10 for details). Green shadings show ± 1 standard deviation of all 18 *r* values per month. Horizontal blue lines approximate the 99% confidence interval after correction for lag1 autocorrelation in the timeseries. The bottom panels are monthly minimum, mean and maximum temperatures exceeding 5˚C that is commonly considered as ‘biological zero’

Seven MXD records from alpine treeline ecotones in Scandinavia, the Tatra mountains, the Alpine arc and Mount Smolikas reveal unimodal correlation patterns with different monthly temperature combinations between April and September (Fig. 2). In contrast, bimodal correlation patterns are exhibited by the two MXD records from the Iberian Peninsula. Correlation coefficients between the MXD records and monthly temperature means of the year before tree growth were found to be insignificant (not shown). Low first-order autocorrelation in all MXD timeseries confirms that the effect of previous year climate is irrelevant, and slightly higher lag-1 values in the two dendro datasets from the Swiss Alps possibly result from the recurring effects of cyclic defoliation by the larch budmoth (*Zeiraphera diniana* or *griseana* Gn.; Baltensweiler and Rubli 1999). The highest correlation coefficients of the seven MXD records from northern and central Europe that show unimodal patterns range from 0.62 to 0.75, whereas those of the two MXD records from the Spanish Pyrenees and Cazorla mountains that show bimodal patterns are 0.75 and 0.52, respectively. In line with earlier reports (Römer et al. 2021), we found overall weaker relationships between monthly temperatures and the Corsican MXD record, for which sampling sites are located below 1600 m asl. The change from unimodal correlations peaking in high summer to a pattern characterized by insignificant correlations between MXD and summer warmth should not be confused with evidence of absence for the physiological importance of high temperatures on xylogenesis.

A closer look at the site-specific correlation patterns suggests that statistically meaningful proxy-target relationships tend to occur when minimum temperatures are above ‘biological zero’ at circa 5° C (see Figs. S1–S10 for details). Though subject to imprecise meteorological measurements that neither reflect local site conditions nor the necessary degree of temporal resolution, our findings provide evidence for a thermal thresholds of tree growth (Körner et al. 2023). The MXD records from Scandinavia and the Swiss Alps exhibit unimodal distributions of significant positive correlations with monthly means between June and August when average minimum temperatures exceed around 5° C. Bimodal correlation patterns exhibited by the MXD data from the Pyrenees and Cazorla imply that year-to-year variation in peak summer heat is irrelevant for ring formation, because it is always warm enough. This interpretation challenges existing studies that described the irrelevance of high summer temperatures for MXD and argued for a complex seasonal course of tree metabolism (Büntgen et al. 2017). A better understanding of what insignificant correlations really mean for xylogenesis requires in situ monitoring of cell division, cell expansion, cell wall thickening, lignification and postmitotic senescence (Cuny et al. 2014). Prolonged bimodal growth-climate response patterns might also be disrupted by ephemeral cold spells after large, sulphur-rich volcanic eruptions that can constrain the lignification process of secondary cell walls even at the highest treeline trees in the Spanish Pyrenees (Piermattei et al. 2020). Another factor that is likely to gain in importance under global warming are severe summer droughts that can disrupt Mediterranean tree growth (Battipaglia et al. 2023). If anthropogenic global warming continues at its current pace (Esper et al. 2024), we can expect some unimodal growth-climate response patterns to become bimodal in the future. Moreover, it is most likely that summer temperature extremes above 40 °C will be reached more frequently and affect tree physiological processes, as well as the functioning and productivity of entire forest ecosystems.

## Conclusion

Motivated by the well-known premise that correlation should not been confused with causation, we re-assessed the seasonal temperature response of ten state-of-the-art MXD records from near treeline ecotones between northern Scandinavia and southern Spain. Our findings suggest that low correlations with high temperatures in bimodal response patterns mainly reflect warm summer conditions at Mediterranean sites where interannual variations in peak warming do not affect climate correlations. We conclude that low growth-climate associations within the growing season do not reject the importance of temperature for xylogenesis, whereas insignificant correlations outside the growing usually refer to dormancy.

## Electronic supplementary material

Below is the link to the electronic supplementary material.

Supplementary Material 1
