# Supplementary material for: Physiological meaning of bimodal tree growth-climate response patterns
Source: Int J Biometeorol. 2024 May 30;68(9):1897–902. doi: 10.1007/s00484-024-02706-5 (PMC11461572; doi:10.1007/s00484-024-02706-5)
Supplement: Supplementary file 1 — Supplementary Material 1 [file 484_2024_2706_MOESM1_ESM.docx]

Supplementary information

Figures S1–S10


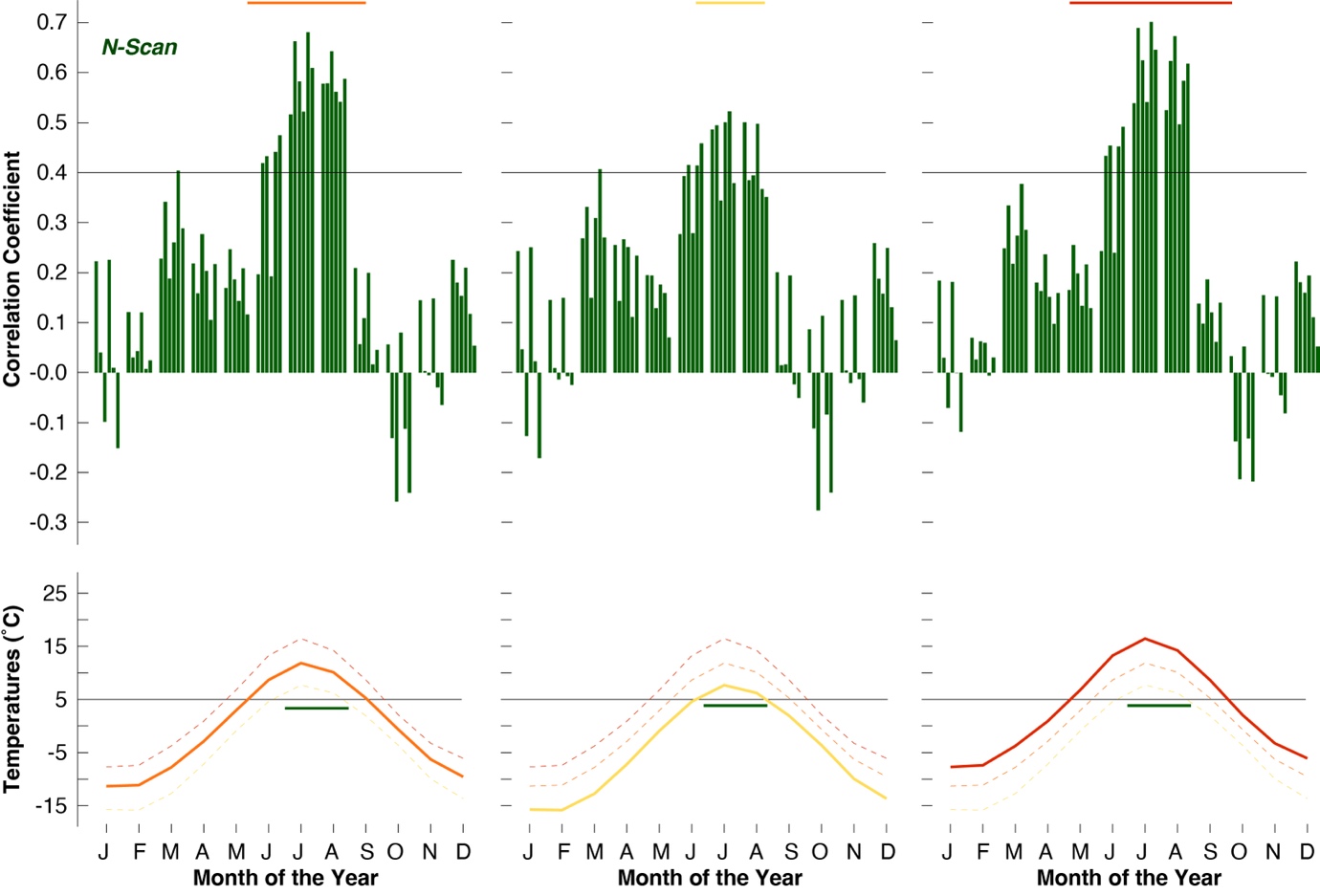


**Figure S1.** Correlation coefficients between the maximum latewood density (MXD) record from northern Scandinavia (N-Scan; Fig. 1) and monthly temperatures from the closest 0.25˚ lat/lon grid box of the latest E-OBS version (v28.0e). Lower panels refer to the corresponding monthly mean, minimum and maximum temperatures (from left to right).


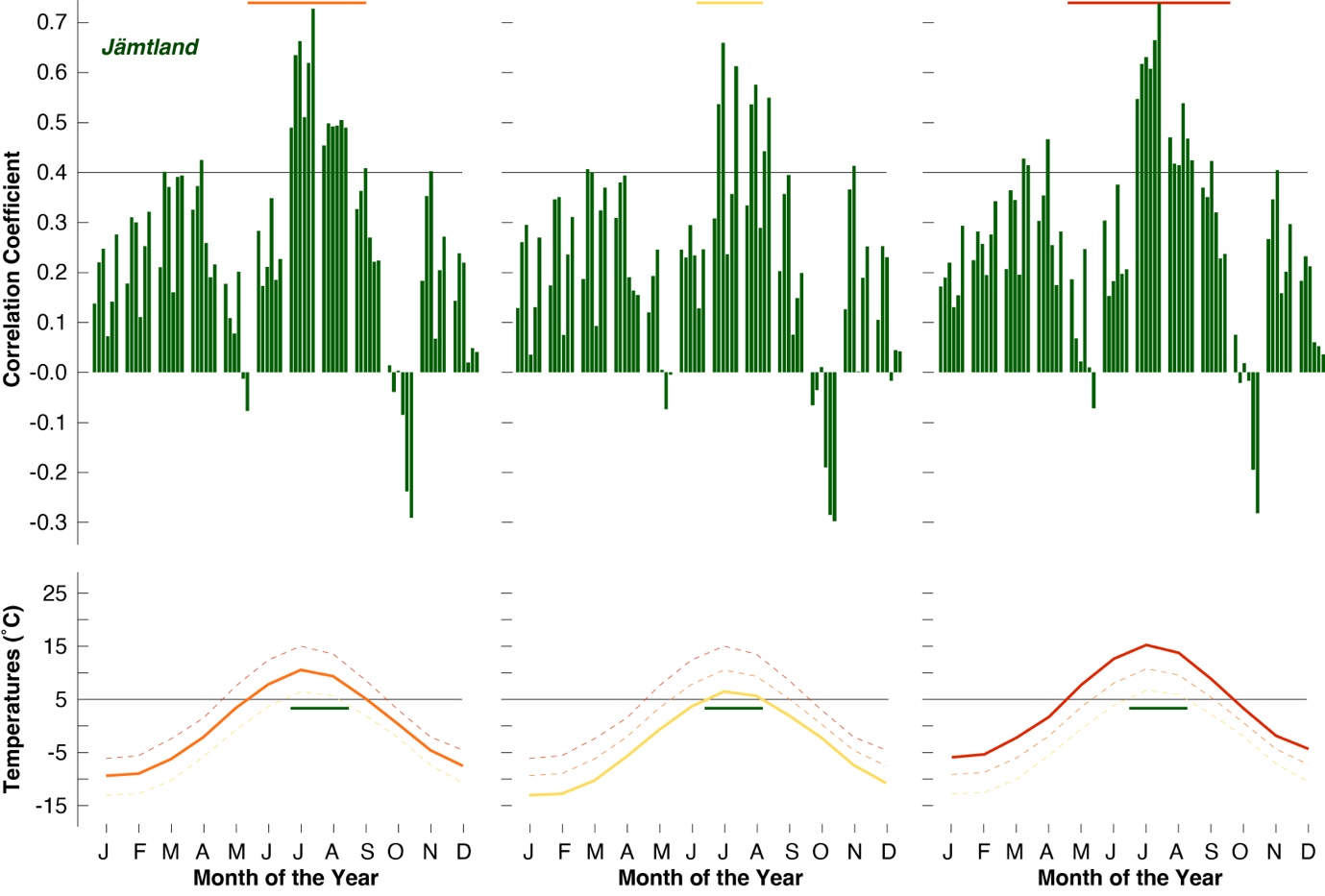


**Figure S2.** Correlation coefficients between the maximum latewood density (MXD) record from central Scandinavia (Jämtland; Fig. 1) and monthly temperatures from the closest 0.25˚ lat/lon grid box of the latest E-OBS version (v28.0e). Lower panels refer to the corresponding monthly mean, minimum and maximum temperatures (from left to right).


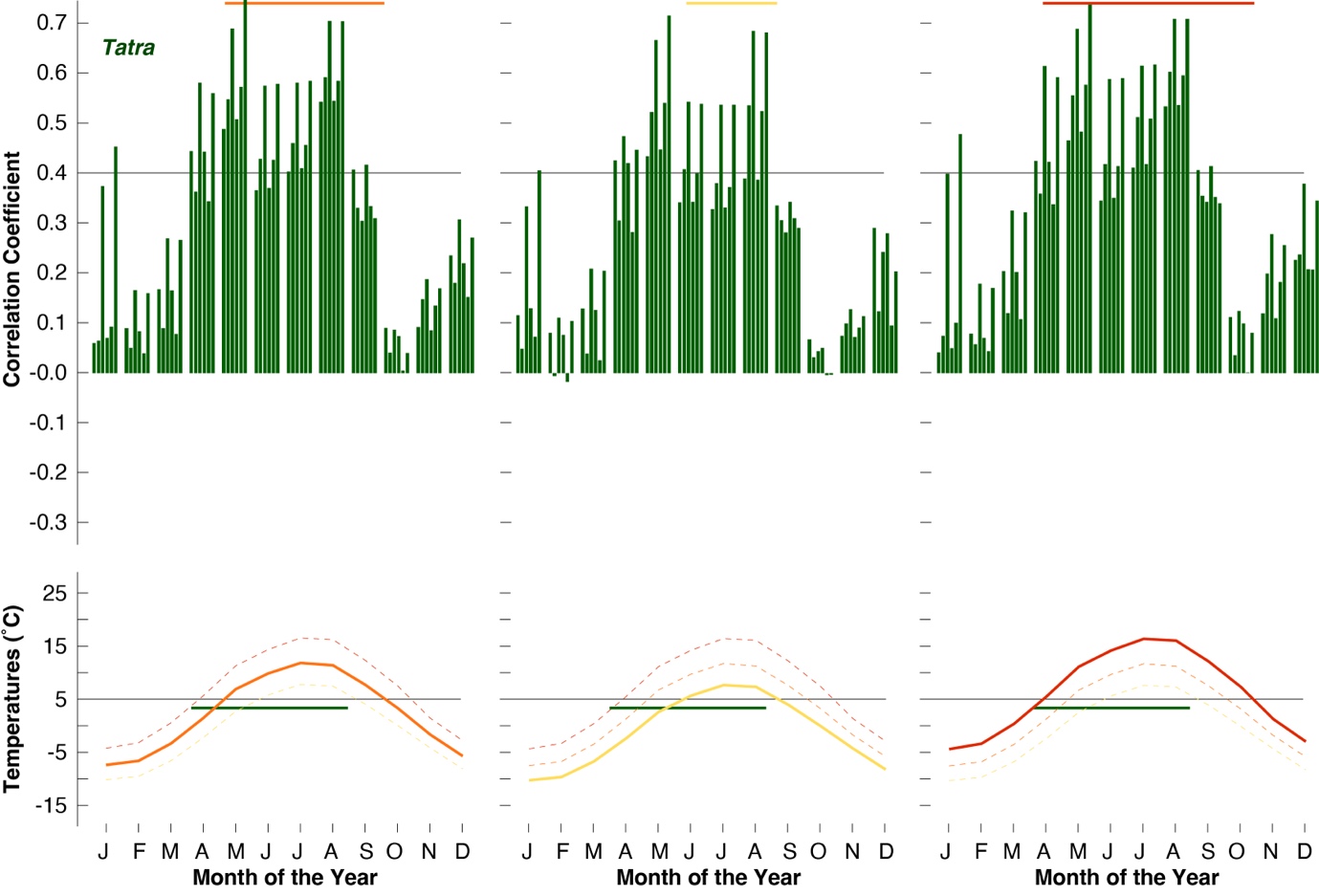


**Figure S3.** Correlation coefficients between the maximum latewood density (MXD) record from the Tatra Mountains (Tatra; Fig. 1) and monthly temperatures from the closest 0.25˚ lat/lon grid box of the latest E-OBS version (v28.0e). Lower panels refer to the corresponding monthly mean, minimum and maximum temperatures (from left to right).


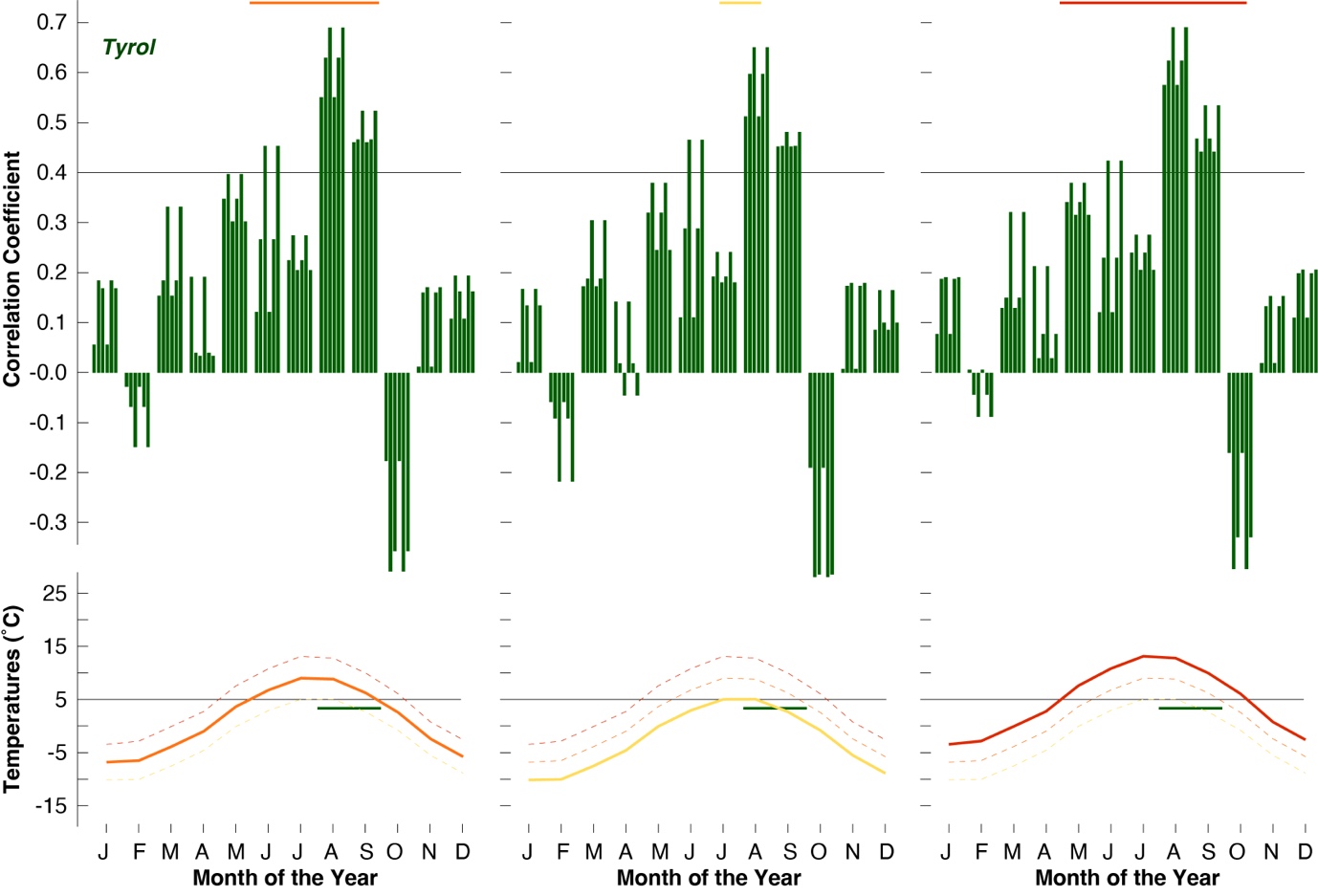


**Figure S4.** Correlation coefficients between the maximum latewood density (MXD) record from the Austrian Alps (Tyrol; Fig. 1) and monthly temperatures from the closest 0.25˚ lat/lon grid box of the latest E-OBS version (v28.0e). Lower panels refer to the corresponding monthly mean, minimum and maximum temperatures (from left to right).


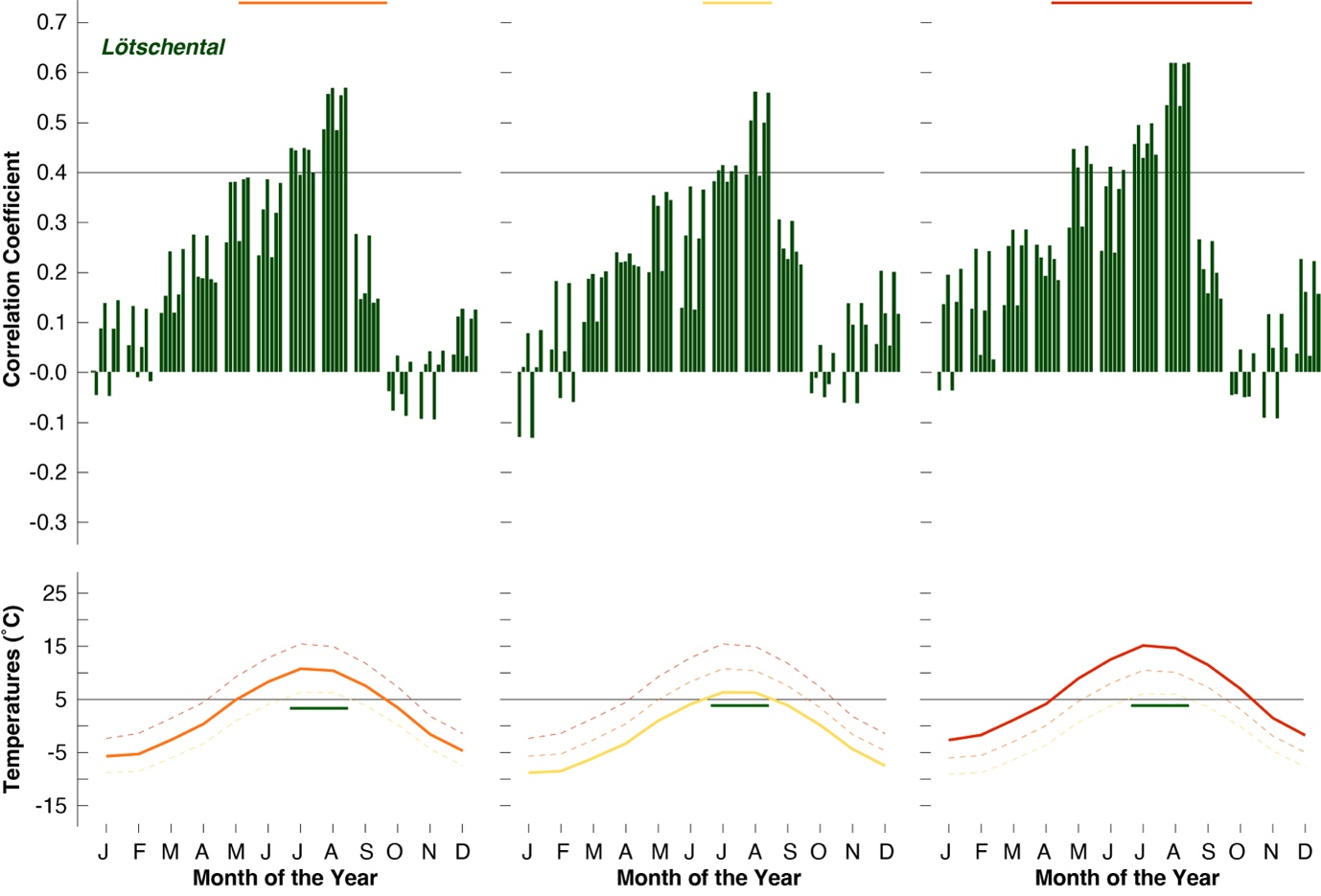


**Figure S5.** Correlation coefficients between the maximum latewood density (MXD) record from the western Swiss Alps (Lötschental; Fig. 1) and monthly temperatures from the closest 0.25˚ lat/lon grid box of the latest E-OBS version (v28.0e). Lower panels refer to the corresponding monthly mean, minimum and maximum temperatures (from left to right).


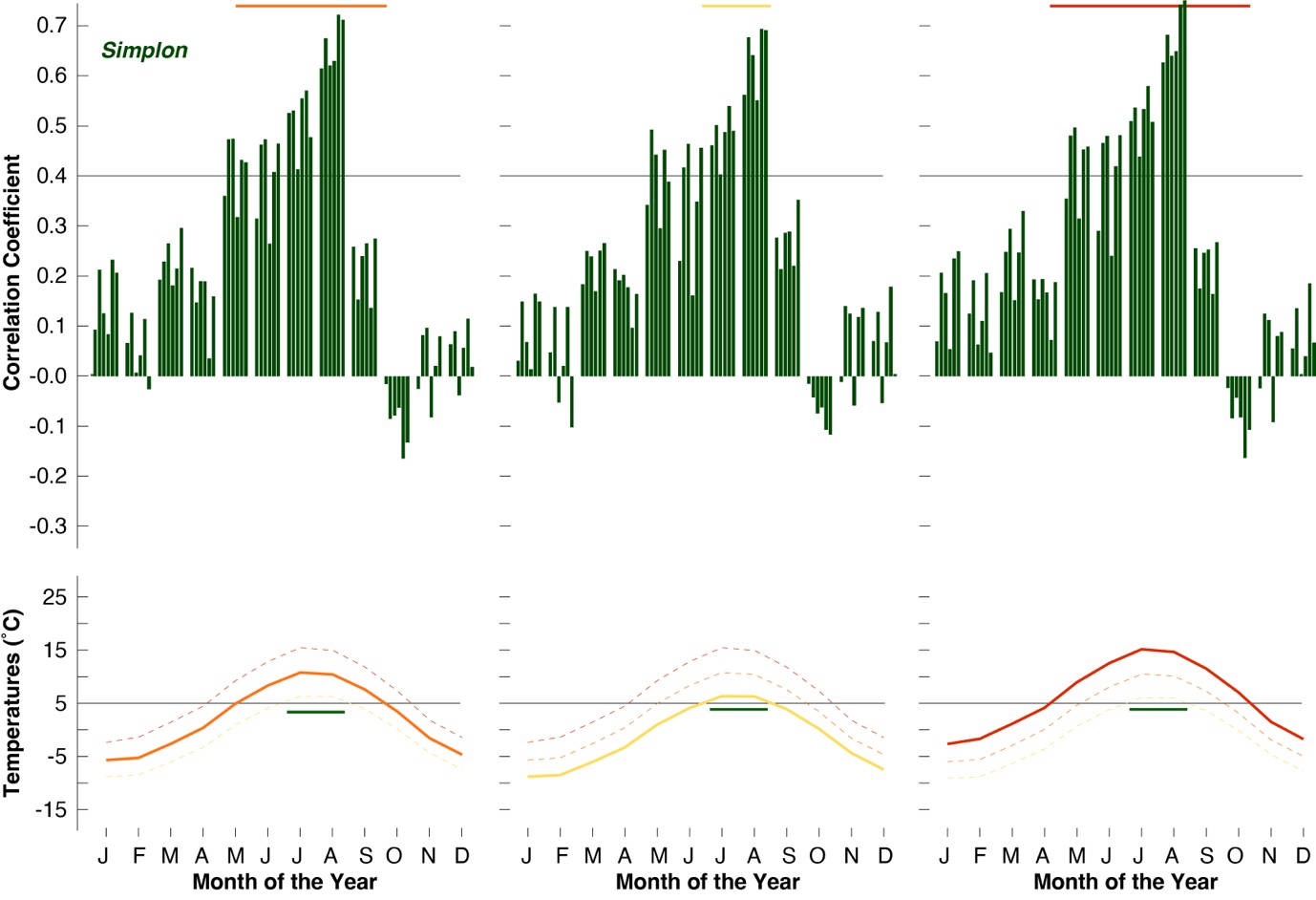


**Figure S6.** Correlation coefficients between the maximum latewood density (MXD) record from southern Swiss Alps (Simplon; Fig. 1) and monthly temperatures from the closest 0.25˚ lat/lon grid box of the latest E-OBS version (v28.0e). Lower panels refer to the corresponding monthly mean, minimum and maximum temperatures (from left to right).

**
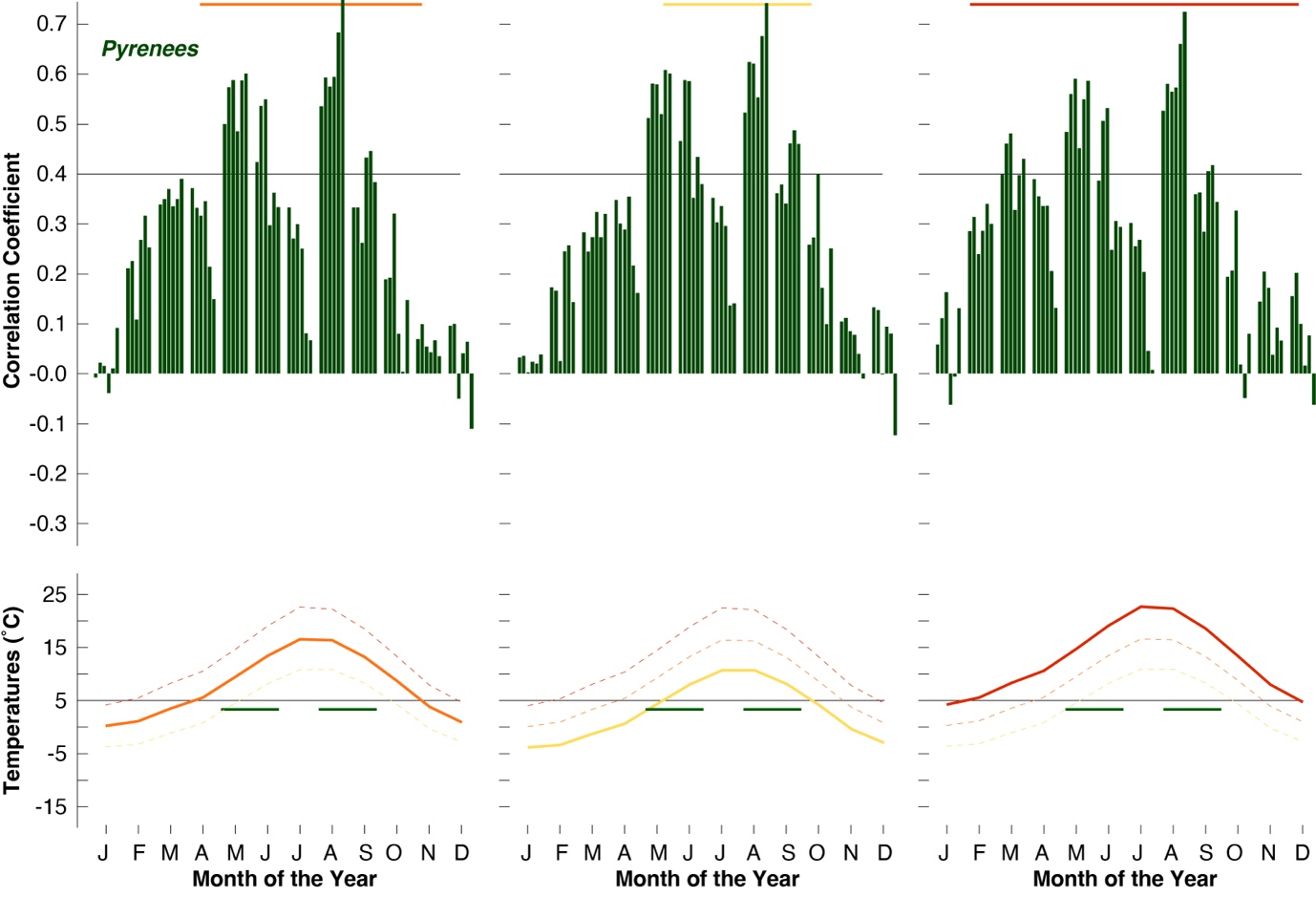
**

**Figure S7.** Correlation coefficients between the maximum latewood density (MXD) record from the central Pyrenees (Pyrenees; Fig. 1) and monthly temperatures from the closest 0.25˚ lat/lon grid box of the latest E-OBS version (v28.0e). Lower panels refer to the corresponding monthly mean, minimum and maximum temperatures (from left to right).


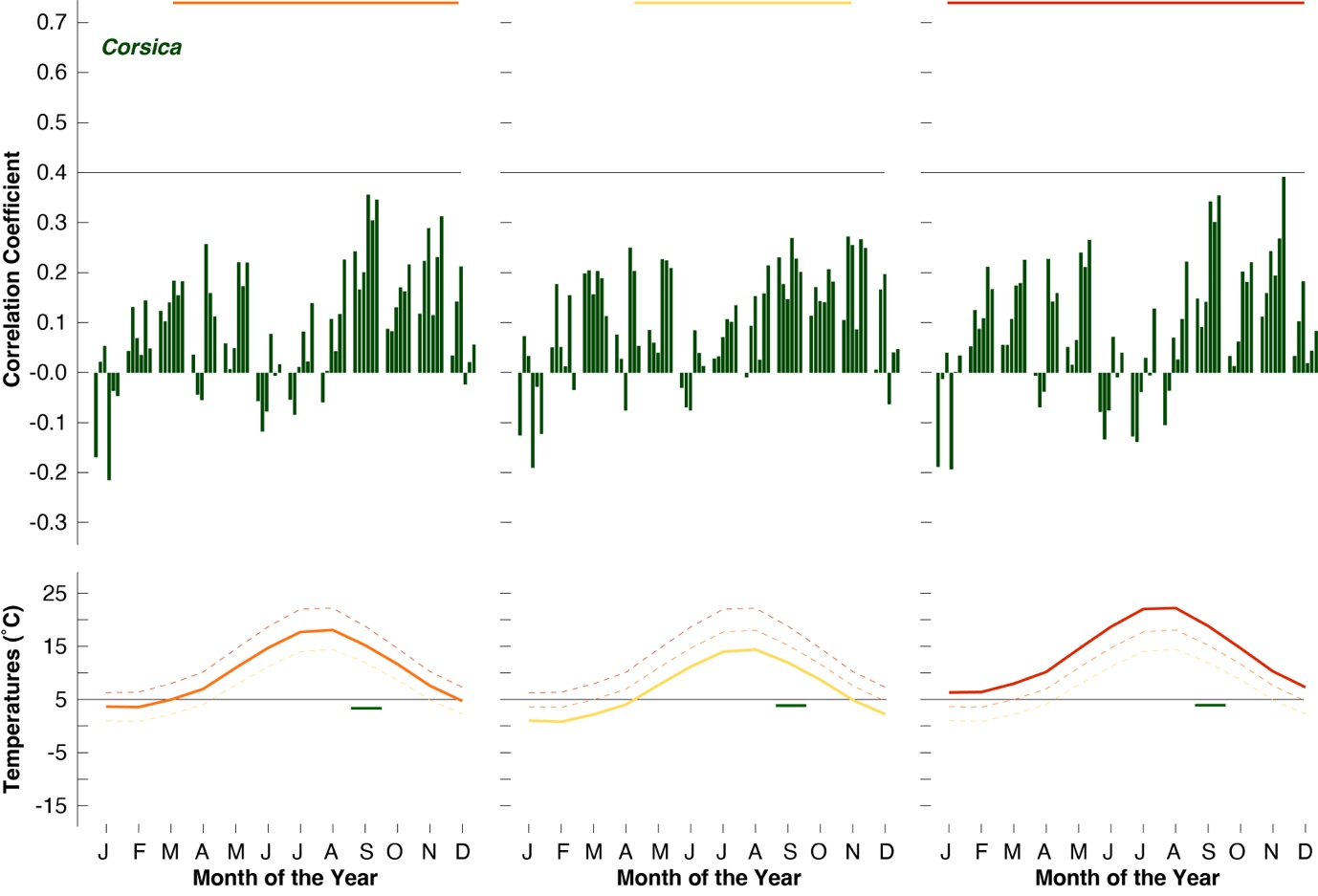


**Figure S8.** Correlation coefficients between the maximum latewood density (MXD) record from northern Corsica (Corsica; Fig. 1) and monthly temperatures from the closest 0.25˚ lat/lon grid box of the latest E-OBS version (v28.0e). Lower panels refer to the corresponding monthly mean, minimum and maximum temperatures (from left to right).


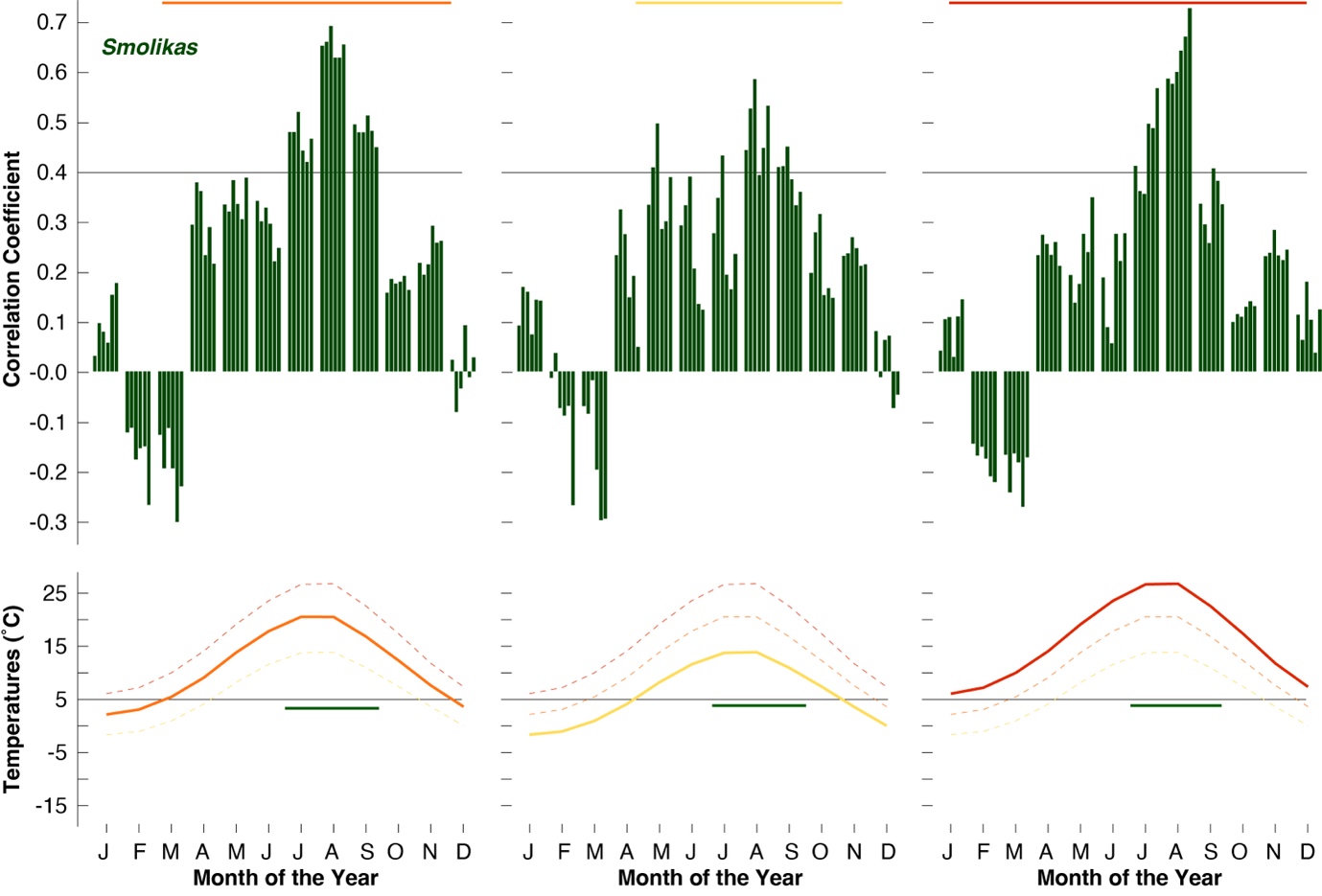


**Figure S9.** Correlation coefficients between the maximum latewood density (MXD) record from northern Greece (Smolikas; Fig. 1) and monthly temperatures from the closest 0.25˚ lat/lon grid box of the latest E-OBS version (v28.0e). Lower panels refer to the corresponding monthly mean, minimum and maximum temperatures (from left to right).


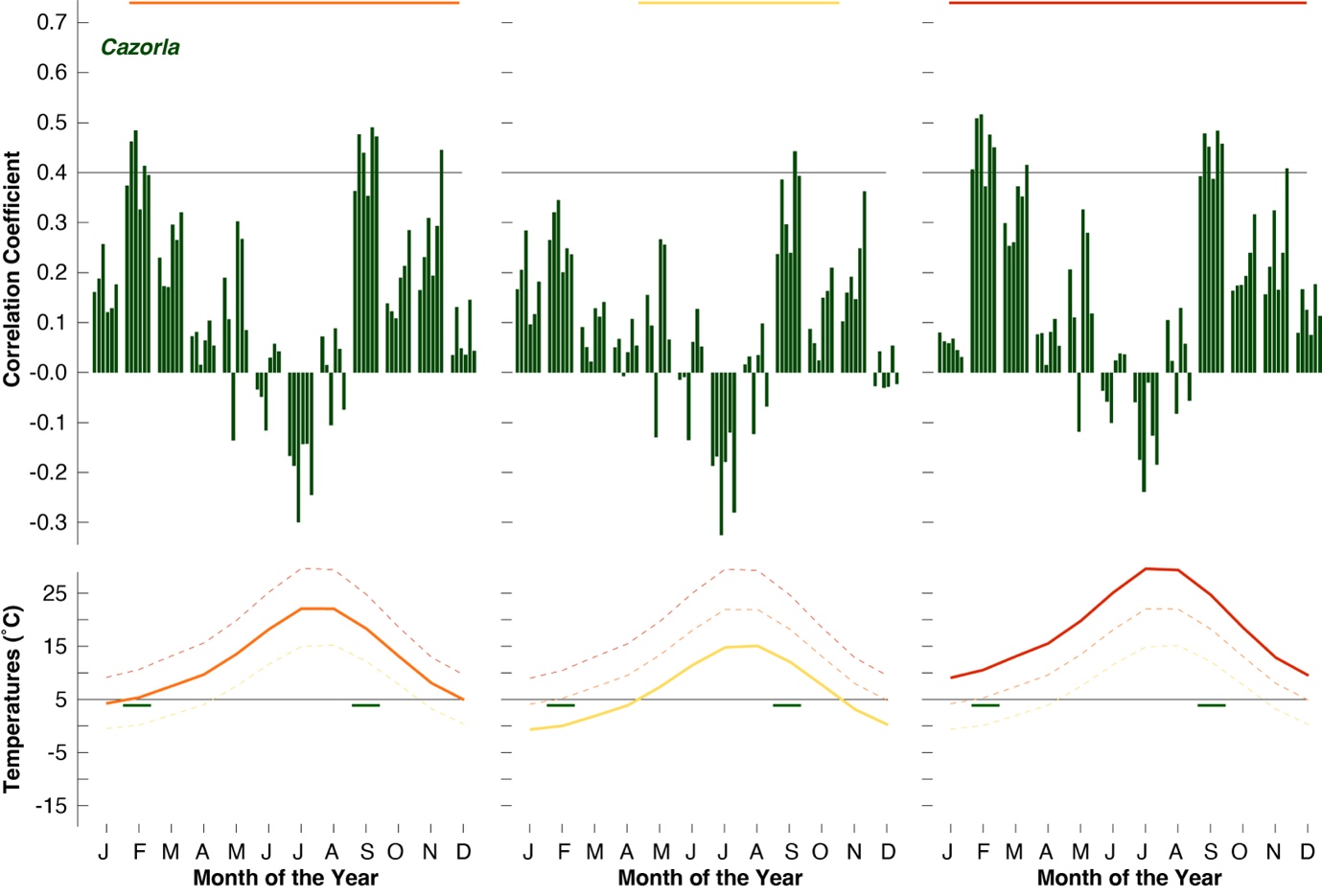


**Figure S10.** Correlation coefficients between the maximum latewood density (MXD) record from southern Spain (Cazorla; Fig. 1) and monthly temperatures from the closest 0.25˚ lat/lon grid box of the latest E-OBS version (v28.0e). Lower panels refer to the corresponding monthly mean, minimum and maximum temperatures (from left to right).
